# Constrained Plug-and-Play Priors for Image Restoration

**DOI:** 10.3390/jimaging10020050

**Published:** 2024-02-19

**Authors:** Alessandro Benfenati, Pasquale Cascarano

**Affiliations:** 1 Environmental and Science Policy Department, University of Milan, Via Celoria 2, 20133 Milano, Italy; 2 Gruppo Nazionale Calcolo Scientifico, INDAM, Piazzale Aldo Moro 5, 00185 Rome, Italy; 3 Department of the Arts, University of Bologna, Via Barberia 4, 40123 Bologna, Italy; pasquale.cascarano2@unibo.it

**Keywords:** plug-and-play priors, constrained formulation, image restoration, inverse problems, regularization by denoising, discrepancy principle

## Abstract

The Plug-and-Play framework has demonstrated that a denoiser can implicitly serve as the image prior for model-based methods for solving various inverse problems such as image restoration tasks. This characteristic enables the integration of the flexibility of model-based methods with the effectiveness of learning-based denoisers. However, the regularization strength induced by denoisers in the traditional Plug-and-Play framework lacks a physical interpretation, necessitating demanding parameter tuning. This paper addresses this issue by introducing the Constrained Plug-and-Play (CPnP) method, which reformulates the traditional PnP as a constrained optimization problem. In this formulation, the regularization parameter directly corresponds to the amount of noise in the measurements. The solution to the constrained problem is obtained through the design of an efficient method based on the Alternating Direction Method of Multipliers (ADMM). Our experiments demonstrate that CPnP outperforms competing methods in terms of stability and robustness while also achieving competitive performance for image quality.

## 1. Introduction

The challenge of reconstructing a high-quality image $x\in R^{n}$ from its degraded measurement $b\in R^{n}$ is commonly formulated as a linear inverse problem. Such a problem has to be addressed in several imaging frameworks, such as in medicine [1,2,3,4], microscopy [5,6,7], and astronomy [8,9,10]. Although these are different and maybe distant topics, they share a common linear model [11] for the image acquisition process: namely,

(1) b=Ax+η,

where $A\in R^{n\times n}$ is a known blur operator called the Point Spread Function (PSF) [12], and $\eta \in R^{n}$ represents additive random noise with a standard deviation of $\sigma_{\eta }$. Linear inverse problems, due to the physics underlying the data acquisition process, often suffer from ill-posedness [12], necessitating the formulation of the solution $x^{★}\in R^{n}$ as a minimizer of a regularized objective function.

The standard model-based approach involves minimizing a regularized objective function of the form:

(2) $$x^{*}\in \underset{x\in R^{n}}{\mathrm{argmin}}\;𝓁\left(x;A,b\right)+\mu \rho \left(x\right),$$

where 𝓁(x;A,b) encodes data fidelity information, and ρ(x) is the regularization term. The choice of the function *ℓ* depends on the statistical noise perturbing the data. In the presence of additive Gaussian noise (AWGN), the natural choice is the least square function, whilst signal-dependent noise requires tailored functionals: for example, Poisson noise induces the employment of the Kullback–Leibler functional [13,14,15]. Defining the regularization parameter μ>0 is crucial, and it is often selected by hand. The imaging community has developed several techniques for the automatic choosing of such a parameter. For example, well established methods such as the discrepancy principle, L-curve, or cross-validation [16] have been considered for Gaussian noise, while the discrepancy principle has been adapted also in the presence of Poisson noise [17,18]. Alternatively, another interesting approach consists of recasting the optimization problem (2) as a constrained one [19,20,21]: namely,

(3) $$x^{*}\in \underset{x\in R^{n}}{\mathrm{argmin}}\;\rho \left(x\right)\;s.t.\;𝓁\left(x;A,b\right)\leq c.$$

The positive scalar *c* represents the strength of the constraints, and different from the parameter μ in (2), it has a physical meaning. For example, the value of *c* in (3) usually depends on the amount of noise.

Another grand challenge for the model-based approach is the design of an effective regularization functional ρ(x) capable of capturing intricate image features. Some examples include the well-known Tikhonov regularization [22], known as ridge regression in statistical contexts, and its variant that promotes diffuse components on the final reconstruction; the total variation functional, which aims to preserve sharp edges [2,23,24]; the ${𝓁}_{p}$-norms regularizers, with 0≤p≤1, which induce sparsity on the image and/or gradient domains [19,25,26]; and the elastic-net functional [4], which is a convex combination of ${𝓁}_{1}$ and ${𝓁}_{2}$ norms.

Nowadays, it has been recognized that among the possible strategies to solve imaging inverse problems, learning-based techniques represent the most efficient alternative. The advancements in deep learning for imaging problems were driven by the application of neural networks to learn an inverse mapping from measurements to the image space to obtain an approximate solution. In particular, a dataset is composed by considering several acquisitions and their corresponding ground truths, and a neural network is trained in order to minimize the empirical risk. This end-to-end approach is very interesting since the forward degradation model (1) is not needed. This property is extremely useful when the problem of interest is physically unknown or hard to express with an analytical expression. Another appealing characteristic of these inverse learning methods is their computational efficiency during the inference, as they outperform standard variational techniques. However, the major limitation of learning approaches regards the stability of the models [27]. The presence of noise in the measurement can produce various artifacts in the reconstruction obtained through a neural network [28]. Moreover, when a measurement falls outside the training set distribution, the model may produce hallucinated reconstructions containing misleading artifacts [29]. Such undesired behavior is particularly problematic in some applications. In addition, different from variational approaches, learned models have to be retrained whenever the acquisition model changes.

A popular technique to construct a model that is adaptable to various imaging tasks with loose dependence on the training data consists of decoupling the degradation model from the learning-based prior. The fast development of learning-based techniques in the field of imaging inverse problems has allowed the defining of data-driven regularizers that have largely outperformed handcrafted ones [30]. This approach is often referred to as Plug-and-Play (PnP) and represents a versatile and innovative paradigm to impose a statistically learned prior within a variational framework. The PnP prior framework [31,32,33,34,35] has emerged as a potent approach that leverages advanced denoisers as regularizers without explicitly defining ρ(x). However, the lack of an explicit objective function complicates theoretical analyses [35]. Regularization by denoising (RED) [36] addresses this by formulating an explicit regularization functional, but practical challenges persist, especially in ensuring denoisers align with the manifold of natural images.

The complexity of selecting an appropriate regularizer prompts exploration beyond traditional handcrafted terms. While model-based approaches often rely on handcrafted terms, this paper advocates for the PnP framework and demonstrates that closed-form regularizers are not always optimal for inducing prior information. The PnP approach, rooted in proximal algorithms, allows substituting the regularization term with off-the-shelf denoisers, diversifying prior information sources. The paper concludes with an overview of existing PnP studies that highlights the modular structure’s flexibility and the versatility of employing various proximal algorithms and denoisers.

The presented work is organized as follows. Section 2 is devoted to provide a brief introduction to the PnP method (Section 2.1), and then it presents the novel constrained approach (Section 2.2). Section 3 addresses the performance of the the proposed model. In particular, we define the implementation settings (Section 3.1); we discuss selection of the optimal denoiser, examining how different denoising priors influence the overall performance of the proposed model (Section 3.2); we show the robustness of the proposed method with respect to its parameters (Section 3.3 and Section 3.4); we provide comparisons from a quantitative and qualitative perspective with similar state-of-the-art algorithms (Section 3.5). Finally, Section 4 draws the conclusion and future perspectives.

### Notations

Bold small letters refer to vectors, while bold capital letters refer to matrices. The operator ${\mathrm{prox}}_{f}\left(x\right)$ stands for the proximity operator of *f*: ${\mathrm{prox}}_{f}\left(x\right)=\underset{y}{\mathrm{argmin}}\;f(y)\;+\;\gamma /2{∥x\;-\;y∥}_{2}^{2}$. The projection on a set A is denoted with ${\mathrm{proj}}_{A}$. Square or rectangular images are vectorized: an image $x\in R^{p\times q}$ is seen as a vector belonging to $R^{n}$, where n=pq and the elements of x are stacked column-wise. The term $\iota_{A}$ denotes the indicator function of the set A. $R^{+}$ denotes the set of positive real numbers.

## 2. Constrained PnP Model

This section briefly introduces the Plug-and-Play approach: showing its main idea and convergence properties. The second part is devoted to presenting the novel constrained strategy.

### 2.1. Plug-and-Play Models: A Brief Overview

The building block of Plug-and-Play methods is the established Alternating Direction of Multipliers Method (ADMM) used to solve Problem (2): this method introduces a novel variable that induces a further constraint; in this way, one is led to solve the following problem.

(4) $$\underset{x=v}{\mathrm{argmin}}\;𝓁\left(x;A,b\right)+\mu \;\rho \left(v\right).$$

The augmented Lagrangian function for (4) reads as

$$L\left(x,v,\mu \right)=𝓁\left(x;A,b\right)+\mu \rho \left(v\right)+\langle \lambda ,x-v\rangle +\frac{\beta }{2}{∥x-v∥}_{2}^{2},$$

where $\beta \in R^{+}$ is a penalty parameter and $\lambda \in R^{n}$ is the Lagrangian parameter relative to the constraint x=v. After minimal algebraic manipulations, the new unconstrained problem to be solved is

(5) $$\min_{x,v\in R^{n}}\max_{\lambda \in R^{n}}𝓁\left(x;A,b\right)+\mu \;\rho \left(v\right)+\frac{\beta }{2}{∥x-v+\frac{\lambda }{\beta }∥}_{2}^{2}-\frac{1}{2}{∥\frac{\lambda }{\beta }∥}^{2}.$$

Such a problem is addressed by the Alternate Direction Method of Multipliers, which is shown in Algorithm 1. The astute reader will recognize that Steps 3 and 4 in Algorithm 1 are the proximity operators [37] of *ℓ* and μρ computed at $v^{k}-\lambda^{k}/\beta$ and at $x^{k+1}+\lambda^{k}/\beta$, respectively. For classical choices for *ℓ*, such as the least square or the Kullback–Leibler functionals, the proximity operators have explicit expressions (see [38] for a comprehensive list of proximity operators for several families of functions). The straightforward expression for the proximity operator of μρ is available for particular regularizations: such as, for example, ${𝓁}_{1}$, whose prox is the soft thresholding operator, and Tikhonov regularization, whose prox is simply a rescaling of the input [38].
**Algorithm 1:** Alternating Direction Method of Multipliers (ADMM).1:Select parameters $\beta ,\mu \in R^{+}$, set $x^{0},v^{0},\lambda^{0}$.2:**for** k=0,1,... **do**3:    $x^{k+1}=\underset{x\in R}{\mathrm{argmin}}\;𝓁\left(x^{k};b\right)+\frac{\beta }{2}∥x-v^{k}+\frac{\lambda^{k}}{\beta }∥$4:    $v^{k+1}=\underset{v\in R}{\mathrm{argmin}}\;\mu \;\rho \left(v\right)+\frac{\beta }{2}∥x^{k+1}-v+\frac{\lambda^{k}}{\beta }∥$5:    $\lambda^{k+1}=\lambda^{k}+\beta \left(x^{k+1}-v^{k+1}\right)$6:**end for**

Under the framework presented in [31], Step 3 of Algorithm 1 is considered as a reconstruction step and provides the maximum a priori estimate given the data b and the operator A. Step 4 is the denoising operator: this depends obviously on the design choice in (2), namely, in the choice for ρ. The strategy depicted in [31] instead suggests bypassing this designing and directly employing a denoising operator *D* in Step 3.

This is tantamount to still considering an objective function as in (2), but regularization functional ρ is *unknown*. Algorithm 2 assumes that the operator *D* is the proximity operator of the unknown function μρ computed at x+λ/β:

$$v=D\left(x+\lambda /\beta \right)={\mathrm{prox}}_{\mu \rho }\left(x+\lambda /\beta \right).$$

After its first presentation to the scientific community, several researcher showed that under a suitable hypothesis, Plug-and-Play methods converge to a solution of the original problem. In [39], the authors showed fixed-point convergence under the usage of denoisers belonging to the bounded denoiser class. The authors in [40] adopt an incremental version of PnP algorithm and prove its convergence under some explicit hypothesis on *ℓ* and on the chosen denoiser. Ref. [41] shows that under the hypotheses of *D* being averaged [41] (Definition 2.1) and *ℓ* being convex, Algorithm 2 converges and, moreover, that it can be proved that some denoisers are actually the prox of some particular functions (e.g., the non-local-mean filter is the prox of a quadratic convex function).
**Algorithm 2:** Plug-and-Play method (PnP).1:Select parameters $\beta \in R^{+}$, set $x^{0},v^{0},\lambda^{0}$, select a denoiser $D:R^{n}\to R^{n}$.2:**for** k=0,1,... **do**3:    $x^{k+1}=\underset{x\in R}{\mathrm{argmin}}𝓁\left(x^{k};b\right)+\frac{\beta }{2}∥x-v^{k}+\frac{\lambda^{k}}{\beta }∥$4:    $v^{k+1}=D\left(x^{k+1}\right)$5:    $\lambda^{k+1}=\lambda^{k}+\beta \left(x^{k+1}-v^{k+1}\right)$6:**end for**

### 2.2. The Proposed Constrained Model

Under the hypothesis of additive noise in (1) and following the constrained approach [42] shown in (3), Problem (2) is reformulated as

$$\underset{x\in R^{n}}{\mathrm{argmin}}\;\rho \left(x\right)\;s.t.\;𝓁\left(x;A,b\right)\leq \delta ,$$

where $\delta =\sqrt{n}\;\tau \;\sigma_{\eta }$, with τ∈[0,1] and $\sigma_{\eta }$ being the known noise level. Hereafter, we assume that an AWGN framework, i.e., additive Gaussian noise is perturbing the image, and hence, the choice of a fit-to-data functional consists of the least square:

$$𝓁\left(x;A,b\right)=\frac{1}{2}{∥Ax-b∥}_{2}^{2}.$$

The constrained formulation, hence, has the following form:

(6) $$\underset{x\in R^{n}}{\mathrm{argmin}}\;\rho \left(x\right)\;\mathrm{subject}\;\mathrm{to}\;\frac{1}{2}{∥Ax-b∥}_{2}^{2}\leq \delta ,$$

We make the following assumption:

**Assumption** **1.** *The function ρ is continuous.*

The constraint set is compact; thus, under Assumption 1, Problem (6) has at least one solution by Weierstrass theorem. Problem (6) can be equivalently recast into the following form:

(7) $$\begin{array}{cc} \; & \underset{x,v,r\in R^{n}}{\mathrm{argmin}}\;\rho \left(x\right)+\iota_{B_{\delta }}\left(r\right),\;s.t\;r=Ax-b,\;x=v, \end{array}$$

where $B_{\delta }:=\{r\in R^{n}\;\left|\;{∥r∥}_{2}^{2}\leq \delta \right\}$ is a closed disk with a zero center and radius δ. Adopting the approach of Section 2.1, the augmented Lagrangian function relative to Equation (7) reads as

(8) $$\begin{array}{cc} L( & x,r,v;\lambda_{v},\lambda_{r})=\;\rho \left(v\right)+i_{B_{\delta }}\left(r\right)\\ \; & +\frac{\beta_{r}}{2}{∥Ax-b-r+\frac{\lambda_{r}}{\beta_{r}}∥}_{2}^{2}\\ \; & +\frac{\beta_{v}}{2}{∥x-v+\frac{\lambda_{v}}{\beta_{v}}∥}_{2}^{2}-\frac{1}{2}{∥\frac{\lambda_{r}}{\beta_{r}}∥}_{2}^{2}-\frac{1}{2}{∥\frac{\lambda_{v}}{\beta_{v}}∥}_{2}^{2}, \end{array}$$

where $\lambda_{r}$ and $\lambda_{v}$ are the Lagrange multipliers, and $\beta_{r}$ and $\beta_{v}$ are the proximity penalties. The new task to be addressed is, hence,

$$\min_{x,v,r}\max_{\lambda_{r},\lambda_{v}}L\left(x,r,v;\lambda_{v},\lambda_{r}\right)$$

and then the PnP-ADMM approach can be adopted to solve the above optimization problem. As previously shown, this amounts to substituting the proximity operator of ρ computed at $x+\lambda_{v}/\beta_{v}$ with a denoiser computed at the same point. Algorithm 3 shows the implementation of this strategy.

The noise level δ may be a priori known; on the other hand, when information only about the type of noise is available, one can find in the literature several methods to estimate the noise level (see for example [43]). The computation of $x^{k+1}$ seems to pose some computational issues since it requires the inversion of a matrix, which could be cumbersome in terms of computational cost. Nonetheless, under a suitable hypothesis for the operator A, which is practically satisfied in real-life applications, such an update can be easily pursued in Fourier spaces via FFTs.

**Remark** **1** (Convergence of the ADMM approach). *We discuss some observations about the convergence of the presented scheme in Algorithm 3.*

- *A well-established result [44] states that ADMM converges even when more than two variables are considered in the formulation.*
- *The substitution of the proximity operator in Step 4 may hinder the convergence behavior of the whole algorithm. Coupling the result from [44] with [41], for example, assures the convergence for a suitable denoiser D.*

**Algorithm 3:** Constrained Plug-and-Play approach (CPnP).
1:Set δ, $x^{0},v^{0}=0$, and $r^{0}=b-Ax^{0}$, select $\beta_{u},\;\beta_{r}>0$, and initialize $\lambda_{r}^{0},\lambda_{v}^{0}$. Choose a denoiser *D*.2:**for** k=0,1,... **do**3:    $x^{k+1/2}=\frac{\beta_{r}}{\beta_{v}}A^{⊤}(b+r^{k}-\frac{\lambda_{r}^{k}}{\beta_{r}})+(v^{k}-\frac{\lambda_{v}^{k}}{\beta_{v}})$4:    $x^{k+1}\leftarrow {\left(\frac{\beta_{r}}{\beta_{v}}A^{⊤}A+I\right)}^{-1}x^{k+1/2}$5:    $v^{k+1}=D(x^{k+1}+\frac{\lambda_{v}^{k}}{\beta_{v}})$6:    $r^{k+1}={\mathrm{proj}}_{B\delta }(Ax^{k+1}-b+\frac{\lambda_{r}^{k}}{\beta_{r}})$7:    $\lambda_{r}^{k+1}\leftarrow \lambda_{r}^{k}+\beta_{r}\left(r^{k+1}-Ax^{k+1}+b\right)$8:    $\lambda_{v}^{k+1}\leftarrow \lambda_{v}^{k}+\beta_{v}(x^{k+1}-v^{k+1})$9:
**end for**

**Remark** **2.** *We point out that the constrained approach has a remarkable positive outcome: it avoids selection of the regularization parameter in Problem (2).*

## 3. Results

In this section, we delve into the outcomes of our study. The first subsection begins with an overview of the methodological setting. This encompasses the experimental setup, the metrics employed for the evaluation of the results, and a comprehensive presentation of the comparative methods. Subsequently, we focus on the analysis of the proposed method with respect to the choice of the ADMM penalty sequences. The final section offers a detailed examination, both qualitatively and quantitatively, of our approach in comparison to its competitors.

### 3.1. Settings, Evaluation Metrics, and Competing Baseline Methods

As a case study, we focus on the task of image deblurring with AWGN assumptions. Accordingly, in Equation (1), A represents a Gaussian blurring operator with a standard deviation of $\sigma_{A}$, and η denotes zero-mean Gaussian noise with a standard deviation of $\sigma_{\eta }$. We generate blurry and noisy data by applying the image formation model (1) to the images from Set5 [45] and Set24 [46], which are referred to as the ground truths (GTs).

Our method is compared with two baselines: (1) the original unconstrained Plug-and-Play model [47] solved via the half-quadratic splitting (HQS) algorithm and (2) the unconstrained RED formulation [36] solved via ADMM. The former is referred to as PnP and the latter is referred to as RED in the following.

We evaluate the quality of restored images using the peak signal-to-noise ratio (PSNR) and structural similarity index (SSIM) metrics: both assess the quality of the reconstruction. PSNR offers a numerical perspective by measuring the ratio between the maximum possible power of a signal and the power of corrupting noise that affects the fidelity of its representation. It quantifies the degree of distortion present in the restored image compared to the original and considers the full range of pixel values. Higher PSNR values typically indicate better-quality reconstruction as they suggest lower levels of distortion. SSIM evaluates the structural similarity between the restored image and the original from a perceptual standpoint. It considers factors such as luminance, contrast, and structure—mimicking human visual perception. SSIM scores closer to 1 indicate greater similarity between the restored and original images and reflect higher perceived quality. Additionally, from a theoretical standpoint, considering a ground truth image x and its blurred and noisy simulated data b, we use $\sigma_{x}:=\frac{{∥Ax-b∥}_{2}}{\sqrt{n-1}}$ as an unbiased estimator of $\sigma_{\eta }$. Thus, we compare the real noise standard deviation $\sigma_{\eta }$ with $\sigma_{x^{*}}$, where $x^{*}$ refers to the output of the algorithms.

We point out that, as one could expect, the quality of the restored images computed using PnP and RED strongly depends on the choice of the regularization parameters (since they consider unconstrained models). In the experiments, the regularization parameters are selected in order to maximize the PSNR metric. We stress that the proposed CPnP automatically selects the strength of the regularization, thus avoiding highly demanding parameter tuning.

Finally, as the stopping criterion, we choose the relative difference of the iterates within a tolerance of $10^{-4}$. The maximum number of iterations is set to 100 for all the methods.

All the experiments are conducted on a PC with an Intel(R) Core(TM) i7-8565U CPU @ 1.80 GHz 1.99 GHz (Intel Corporation, Santa Clara, CA, USA), running Windows 10 Pro and MATLAB 2023b. The codes of the proposed CPnP are available at https://github.com/AleBenfe/CPNP (accessed on 25 January 2024).

### 3.2. On the Choice of the Denoiser

In this section, we evaluate the CPnP method’s efficacy: specifically, by analyzing its performance when considering various state-of-the-art denoising techniques as denoising engines. Our investigation encompasses the Block-Matching and 3D Filtering (BM3D) [48], Non-Local Means (NLM) [49], and Deep Convolutional Neural Network (DnCNN) [47] methods.

BM3D [48] employs a multi-step process wherein it partitions the image into blocks, identifies similar blocks, collaboratively filters them to estimate clean signals, applies 3D transform filtering to reduce noise, and aggregates these filtered blocks to produce the final denoised image. In contrast, NLM [49] compares local image patches, averages similar patches to estimate clean pixels, and then outputs a denoised image. Lastly, DnCNN [47] utilizes a deep neural network to directly map noisy images to denoised counterparts, utilizing residual learning to boost its performance.

In this section, the focus is on images belonging to the Set5 dataset. We simulate blurred and noisy data by employing the linear image formation model defined in Equation (1) with parameters set to ($\sigma_{A}=0.8$, $\sigma_{\eta }=15$). Through this setup, we aim to assess how each of the aforementioned denoising methods impacts the quality of the restored images with respect to PSNR and SSIM metrics and visual quality.

In Table 1, we report the mean values of PSNR and SSIM for images in the Set5 dataset for NLM, BM3D, and DnCNN. DnCNN consistently demonstrates superior performance compared to both BM3D and NLM across both PSNR and SSIM metrics. Specifically, this suggests that DnCNN induces better regularization compared to BM3D and NLM.

**Figure 1:**
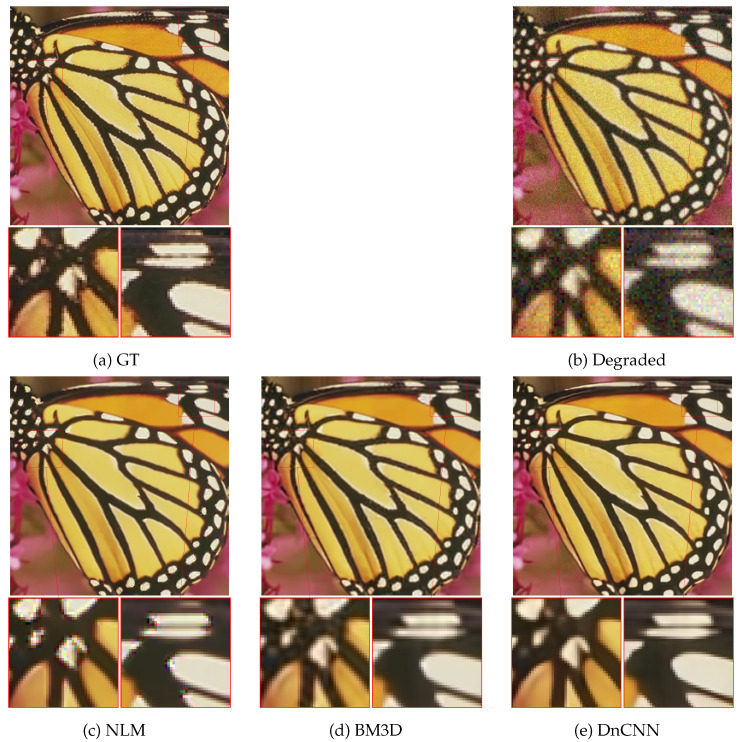
Restoration of the *Butterfly* image from Set5 obtained when selecting different denoising engines: namely, NLM, BM3D, and DnCNN. From left to right: two close-ups of ground truth, degraded image, and the CPnP restorations with NLM, BM3D, and DnCNN. Our CPnP provides more reliable restorations characterized by enhanced clarity and reduced noise when using DnCNN as the denoiser.

In Figure 1, we report the restored images using DnCNN, BM3D, and NLM alongside their respective ground truth and corrupted input data. DnCNN (Figure 1e) produces reconstructions that are noticeably more accurate and clearer (less blurred) and with fine details better preserved. In contrast, NLM (Figure 1c) tends to overly smooth out details, while the BM3D reconstruction (Figure 1d) appears out of focus.

These observations align with the findings from Table 1. The visual assessment further reinforces the superiority of DnCNN and highlights its ability to preserve image details and enhance overall image clarity when compared to traditional denoising methods like NLM and BM3D. Based on these findings, we solely consider DnCNN as the embedded denoiser in our CPnP framework for the subsequent sections.

### 3.3. On the Choice of the Penalty Sequence for the Proposed CPnP

In this section, we investigate the performance of the implemented CPnP method by systematically varying the ADMM penalty sequences. We define increasing penalty sequences according to the relations:

$$\beta_{r}^{k+1}=\gamma \cdot \;\beta_{r}^{k},$$

$$\beta_{v}^{k+1}=\;\gamma \cdot \beta_{v}^{k},$$

where γ≥1. Our experimental setup involves initializing the pair of parameters $\left(\beta_{r}^{0},\beta_{t}^{0}\right)$ from the set ${\left\{0.2,0.4,0.6,0.8,1\right\}}^{2}$ and testing different values of γ: specifically, γ=1,1.01,1.05.

We set τ=1, and for the purposes of our experiments, we assume a known level of noise denoted by $\sigma_{\eta }$. Further discussion on the rationale behind choosing these parameters will be provided in the subsequent section.

As discussed in the previous section, we adopt the DnCNN introduced in [47] as the denoising prior due to its state-of-the-art performance in the field and its fast computation.

We consider the sole *butterfly* image from Set5. We simulate a degraded acquisition by setting $\sigma_{A}=1.2$ and $\sigma_{\eta }=30$. We investigate the stability of CPnP with respect to the choice of γ, $\beta_{r}^{0}$, and $\beta_{t}^{0}$. We point out that in the experiments, we did not observe any significant difference when choosing different images.

**Figure 2:**
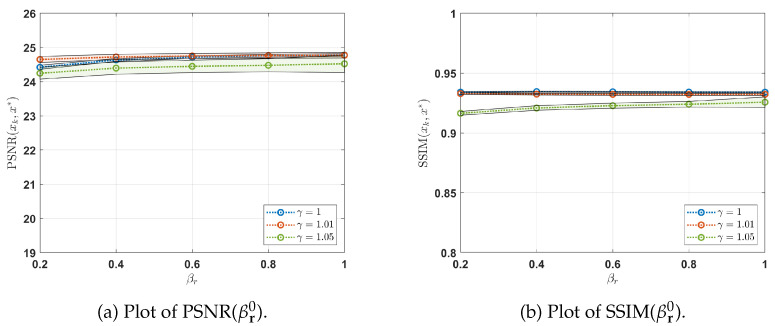
Distribution of PSNR (**a**) and SSIM (**b**) by varying the starting points ($\beta_{r}^{0}$, $\beta_{t}^{0}$) of the increasing ADMM penalty sequences for different value of γ. In (**a**,**b**), for each $\beta_{r}^{0}$, we present the PSNR and SSIM distributions (average ± standard deviation), respectively, with respect to $\beta_{t}^{0}$. The solid lines represent the means of these distributions. The results demonstrate the stability of CPnP and reveal comparable performance in terms of PSNR and SSIM across various γ values and initializations of $\beta_{r}^{0}$ and $\beta_{t}^{0}$.

The stability of the implemented CPnP is depicted in Figure 2a,b. The figures illustrate the distribution of the PSNR and SSIM metrics, respectively, while varying the starting points ($\beta_{r}^{0}$, $\beta_{t}^{0}$) of the increasing ADMM penalty sequences for different values of γ. More specifically, for each $\beta_{r}^{0}$, we consider the distribution of the considered metrics (average ± standard deviation) with respect to $\beta_{t}^{0}$ for different values of γ. In-depth analysis reveals that CPnP consistently maintains high performance levels, as assessed by PSNR and SSIM metrics, even when varying the parameter γ and employing different initializations for $\beta_{r}^{0}$ and $\beta_{t}^{0}$. This robustness across different settings underscores the reliability and effectiveness of the CPnP approach for deblurring and denoising tasks. For all subsequent sections, we set $\beta_{r}^{0}=1$, $\beta_{t}^{0}=1$, and γ=1.01 as fixed parameters.

### 3.4. On the Choice of the Constraint Parameters for the Proposed CPnP

In this section, we consider the images belonging to Set5. We generate blurred and noisy data by applying the linear image formation model (1) with two different degradation settings ($\sigma_{A}=1$, $\sigma_{\eta }=15$) and ($\sigma_{A}=1.3$, $\sigma_{\eta }=30$). We examine the impact on the restored images of $\delta =\tau \;\sqrt{n}\;\sigma_{\eta }$ with τ ranging in [0,1]. Two distinct scenarios are taken into account. In the first, which is referred to as the ideal scenario in the following, we assume that the magnitude of $\sigma_{\eta }$ is exactly known. In the second, which is referred to as the realistic scenario in the following, we assume that only an estimate $\bar{\sigma_{\eta }}$ of $\sigma_{\eta }$ is provided. The estimation is computed following the approach outlined in [43].

**Figure 3:**
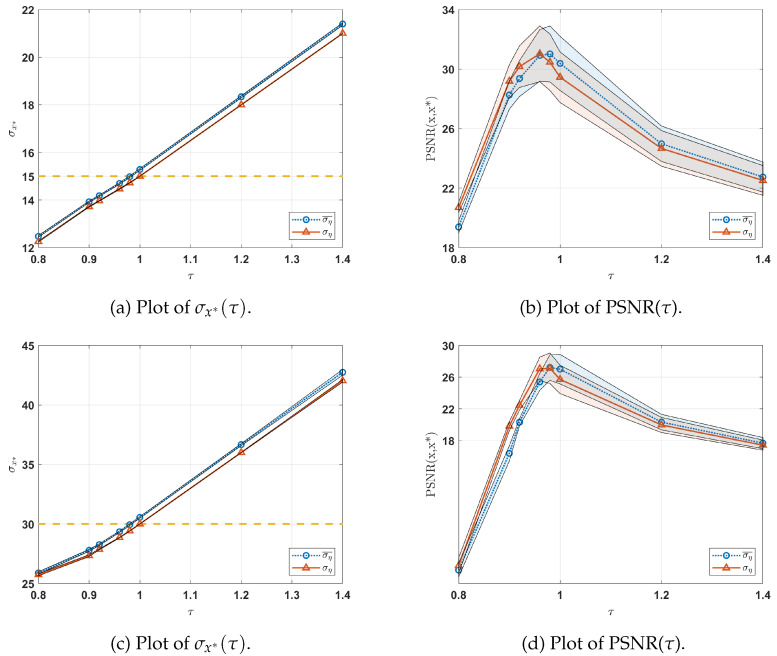
Distribution of $\sigma_{x^{*}}$ (**a**) and PSNR (**b**) by varying τ for the ideal (red line) and realistic (blue line) scenarios. In (**a**,**c**), the dashed yellow line represents the standard deviation of the Gaussian noise corrupting the degraded data ($\sigma_{\eta }=15$ and $\sigma_{\eta }=30$, respectively). In (**a**–**d**), for each τ, we present the $\sigma_{x^{*}}$ and PSNR distributions (average ± standard deviation) with respect to all the images in Set5 for the two different degradation levels considered. The solid red and blue lines represent the means of these distributions.

In Figure 3a–d, we illustrate the variations in $\sigma_{x^{*}}$ and PSNR as a function of τ for the two different degradation levels considered. For each τ, we display the distribution (average ± standard deviation) across all images in Set5 (depicted as shaded regions). The red and blue lines denote the means of these distributions for the idealized and realistic scenarios, respectively. Additionally, in Figure 3a,c, the yellow dashed line represents the true standard deviations (namely $\sigma_{\eta }=15$ and $\sigma_{\eta }=30$) of the Gaussian noise affecting all the data in Set5.

In Figure 3a,c, the idealized scenario (blue lines) demonstrates that the computed $\sigma_{x^{*}}$ closely aligns with $\sigma_{\eta }$ when τ=1 across all images in Set5 (thin shading). In contrast, within the realistic scenario, where only an estimate $\bar{\sigma_{\eta }}$ of the Gaussian noise level is provided, the optimal approximation of $\sigma_{\eta }$ is achieved at τ=0.98 for both degradation levels. Multiple experiments have consistently revealed that this phenomenon stems from the algorithm in [43], which tends to overestimate the noise level in the simulated data b.

Figure 3b,d illustrate the behavior of the PSNR metric with respect to τ. Notably, comparable performance in terms of PSNR is observed for both scenarios. Additionally, Figure 3b,d emphasizes that the highest PSNR is achieved when setting τ<1.

Lastly, Figure 3a indicates that smaller values of τ tend to underestimate, while larger values of τ tend to overestimate $\sigma_{\eta }$. Figure 3b shows that the PSNR metric is negatively affected by these under/overestimations of the noise level.

**Figure 4:**
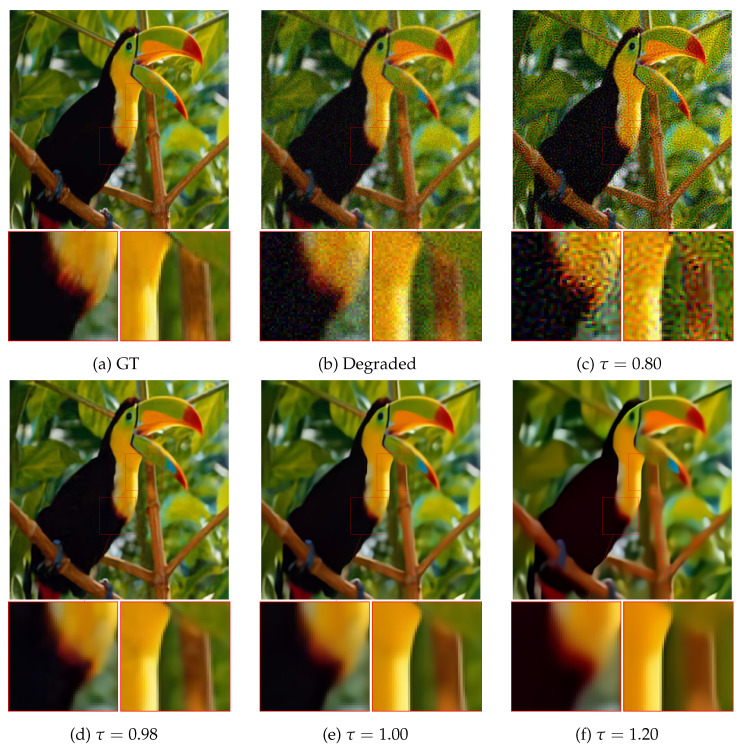
Restoration of the *Bird* image obtained with CPnP for different values of τ. From left to right: two close-ups of ground truth, τ=0.80, τ=0.96, τ=0.98, τ=1.00, and τ=1.20. Small values of τ produce several artifacts on the restored images, whilst large values induce smoothing of the result. The optimal value for τ is close to one; due to the overestimation of the noise level given by [43], τ has to be set strictly less than 1.

Figure 4 provides a visual inspection of the behavior of CPnP with respect to τ values. Small values produce several artifacts in the recovered images, while higher ones induce an oversmoothing effect on the final result. The most reliable results are obtained for τ close to $1^{-}$, i.e., for values close to 1 but still smaller than 1. This behavior is due to overestimation of the noise level given by the employed algorithm [43].

To enhance the reliability of our CPnP testing, we employ the noise level estimation method proposed by [43] to estimate $\sigma_{\eta }$, and consistently set τ=0.98 for all subsequent experiments.

### 3.5. Comparisons with PnP and RED

**Figure 5:**
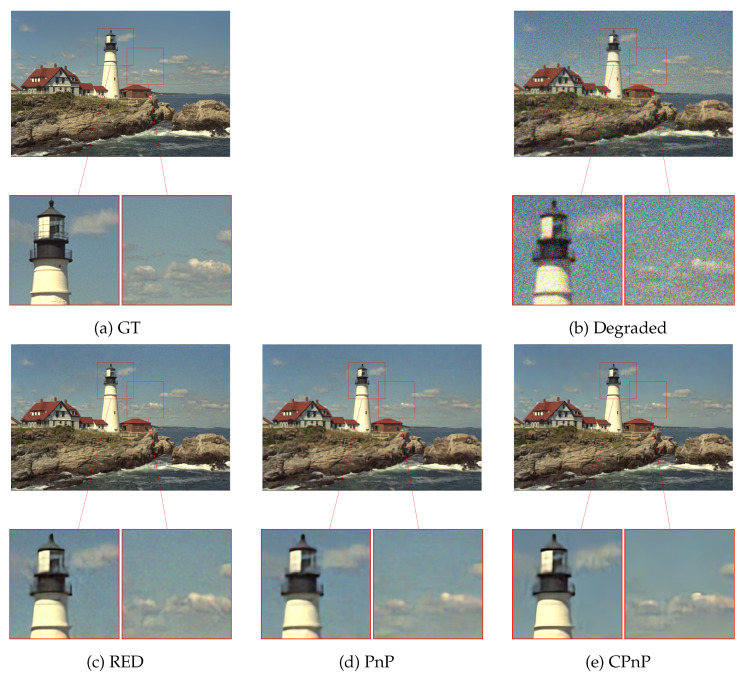
Restoration of the *kodim21* image from Set24 obtained with different methods. From left to right: two close-ups of ground truth, degraded image, RED, PnP, and CPnP. Our CPnP provides more reliable restorations characterized by enhanced clarity and reduced noise.

In this section, we conduct a comparative analysis between our CPnP method and the other two competing methods: PnP and RED. The assessment of reconstruction metric performance involves utilizing images from Set24 under different degradation levels: ($\sigma_{A}=1.2,\sigma_{\eta }=25$) and ($\sigma_{A}=0.8,\sigma_{\eta }=35$). Table 2 presents the mean PSNR and SSIM values. For the competing methods, PnP and RED, the regularization parameters are estimated to achieve optimal performance in terms of the PSNR metric. The results clearly indicate that our CPnP method outperforms both RED and PnP in terms of both performance measures. Furthermore, the effectiveness of our CPnP approach is also assessed from a visual perspective. Figure 5 demonstrates the superiority of CPnP with respect to RED and PnP by providing two close-up views of the restored images. These close-ups highlight the superior reconstruction capabilities of CPnP in terms of clarity and noise reduction compared to both PnP and RED. This visual evidence confirms the outcomes of the quantitative results presented in Table 2 and emphasizes the enhanced performance and reliability of CPnP for deblurring and denoising tasks. Moreover, we finally remark that CPnP exhibits robustness regarding the choice of hyperparameters, unlike RED and PnP, as underlined in the previous sections. The physical interpretation of CPnP hyperparameters enhances the interpretability and practicality of CPnP compared to traditional methods such as RED and PnP.

## 4. Conclusions

In this paper, a novel constrained formulation of the well-established Plug-and-Play framework is presented and is denoted as CPnP. Within this model, the minimum of the regularization functional is compelled to adhere to a discrepancy-based threshold. The solution to the CPnP model is obtained within the ADMM framework and offers a straightforward yet effective approach for image restoration while allowing the usage of different denoising priors.

The determination of the threshold, serving as the regularization parameter, holds physical significance and involves estimating the standard deviation of the noise affecting the data. Efficient assessment of the noise level in the degraded data is achieved through the method outlined in [43] and eliminates the need for extensive parameter tuning as required by unconstrained models like PnP and RED.

In the experimental section, CPnP demonstrates stability and robustness concerning both the model and algorithm hyperparameters. Furthermore, it performs comparably, if not better, than both PnP and RED in terms of PSNR and SSIM metrics as well as visual inspection. The superior performance, coupled with its stability and robustness, position CPnP as a promising choice for various image restoration applications.

## Figures and Tables

**Table 1:** Mean values of PSNR and SSIM for the images in Set5 by varying the embedded denoiser: namely, NLM, BM3D, and DncNN. The best results are highlighted in bold. DnCNN outperforms both BM3D and NLM in terms of the considered metrics.

	Set5 ($\sigma_{A}=0.8,\sigma_{\eta }=15$)		
**Metric**	**NLM**	**BM3D**	**DnCNN**
PSNR	30.27	31.14	**31.52**
SSIM	0.90	0.91	**0.92**

**Table 2:** Mean values of PSNR and SSIM for the images in Set5 and Set24 when varying the degradation levels. The best results are highlighted in bold. Our CPnP outperforms both RED and PnP in terms of the considered metrics.

	Set24 ($\sigma_{A}=1.2,\sigma_{\eta }=25$)			Set24 ($\sigma_{A}\;=\;0.8,\sigma_{\eta }\;=\;35$)		
**Metric**	**RED**	**PnP**	**CPnP**	**RED**	**PnP**	**CPnP**
PSNR	26.29	26.70	**26.85**	26.59	26.92	**27.16**
SSIM	0.75	0.77	**0.78**	0.76	0.77	**0.79**

## Data Availability

The code is available at https://github.com/AleBenfe/CPNP (accessed on 25 January 2024).

## References

[1] Sapienza D., Franchini G., Govi E., Bertogna M., Prato M. (2022). Deep Image Prior for medical image denoising, a study about parameter initialization. Front. Appl. Math. Stat..

[2] Cascarano P., Sebastiani A., Comes M.C., Franchini G., Porta F. Combining Weighted Total Variation and Deep Image Prior for natural and medical image restoration via ADMM. Proceedings of the 2021 21st International Conference on Computational Science and Its Applications (ICCSA).

[3] Coli V., Piccolomini E.L., Morotti E., Zanni L. (2021). A fast gradient projection method for 3D image reconstruction from limited tomographic data. J. Physics Conf. Ser..

[4] Benfenati A., Causin P., Lupieri M., Naldi G. (2020). Regularization Techniques for Inverse Problem in DOT Applications. J. Phys. Conf. Ser..

[5] Calisesi G., Ghezzi A., Ancora D., D’Andrea C., Valentini G., Farina A., Bassi A. (2022). Compressed sensing in fluorescence microscopy. Prog. Biophys. Mol. Biol..

[6] Benfenati A. (2022). upU-Net Approaches for Background Emission Removal in Fluorescence Microscopy. J. Imaging.

[7] Cascarano P., Comes M.C., Sebastiani A., Mencattini A., Loli Piccolomini E., Martinelli E. (2022). DeepCEL0 for 2D single-molecule localization in fluorescence microscopy. Bioinformatics.

[8] Štěpán J., del Pino Alemán T., Bueno J.T. (2022). Novel framework for the three-dimensional NLTE inverse problem. Astron. Astrophys..

[9] Benfenati A., La Camera A., Carbillet M. (2016). Deconvolution of post-adaptive optics images of faint circumstellar environments by means of the inexact Bregman procedure. A&A.

[10] Conroy K.E., Kochoska A., Hey D., Pablo H., Hambleton K.M., Jones D., Giammarco J., Abdul-Masih M., Prša A. (2020). Physics of eclipsing binaries. V. General framework for solving the inverse problem. Astrophys. J. Suppl. Ser..

[11] Bertero M., Boccacci P. (2020). Introduction to Inverse Problems in Imaging.

[12] Bertero M., Boccacci P., Ruggiero V. (2018). Inverse Imaging with Poisson Data.

[13] Benfenati A., Ruggiero V. (2015). Image regularization for Poisson data. J. Phys. Conf. Ser..

[14] Di Serafino D., Landi G., Viola M. (2021). Directional TGV-based image restoration under Poisson noise. J. Imaging.

[15] Bevilacqua F., Lanza A., Pragliola M., Sgallari F. (2023). Whiteness-based parameter selection for Poisson data in variational image processing. Appl. Math. Model..

[16] Bertero M. (2006). Regularization methods for linear inverse problems. Inverse Problems: Lectures Given at the 1st 1986 Session of the Centro Internazionale Matematico Estivo (CIME) Held at Montecatini Terme, Italy, May 28–June 5 1986.

[17] Zanni L., Benfenati A., Bertero M., Ruggiero V. (2015). Numerical methods for parameter estimation in Poisson data inversion. J. Math. Imaging Vis..

[18] Bevilacqua F., Lanza A., Pragliola M., Sgallari F. (2021). Nearly exact discrepancy principle for low-count Poisson image restoration. J. Imaging.

[19] Mylonopoulos D., Cascarano P., Calatroni L., Piccolomini E.L. (2022). Constrained and unconstrained inverse Potts modelling for joint image super-resolution and segmentation. Image Process. Line.

[20] Cascarano P., Franchini G., Kobler E., Porta F., Sebastiani A. (2023). Constrained and unconstrained deep image prior optimization models with automatic regularization. Comput. Optim. Appl..

[21] Wen Y.W., Chan R.H. (2011). Parameter selection for total-variation-based image restoration using discrepancy principle. IEEE Trans. Image Process..

[22] Golub G.H., Von Matt U. (1997). Tikhonov Regularization for Large Scale Problems. Scientific Computing: Proceedings of the Workshop, Hong Kong, 10–12 March 1997.

[23] Campagna R., Crisci S., Cuomo S., Marcellino L., Toraldo G. (2017). Modification of TV-ROF denoising model based on Split Bregman iterations. Appl. Math. Comput..

[24] Rudin L.I., Osher S. Total variation based image restoration with free local constraints. Proceedings of the 1st International Conference on Image Processing.

[25] Lingenfelter D.J., Fessler J.A., He Z. (2009). Sparsity regularization for image reconstruction with Poisson data. Computational Imaging VII.

[26] Cascarano P., Calatroni L., Piccolomini E.L. (2021). Efficient *ℓ*^0^ Gradient-Based Super-Resolution for Simplified Image Segmentation. IEEE Trans. Comput. Imaging.

[27] Szegedy C., Zaremba W., Sutskever I., Bruna J., Erhan D., Goodfellow I., Fergus R. (2013). Intriguing properties of neural networks. arXiv.

[28] Gottschling N.M., Antun V., Adcock B., Hansen A.C. (2020). The troublesome kernel: Why deep learning for inverse problems is typically unstable. arXiv.

[29] Antun V., Renna F., Poon C., Adcock B., Hansen A.C. (2020). On instabilities of deep learning in image reconstruction and the potential costs of AI. Proc. Natl. Acad. Sci. USA.

[30] Arridge S., Maass P., Öktem O., Schönlieb C.B. (2019). Solving inverse problems using data-driven models. Acta Numer..

[31] Venkatakrishnan S.V., Bouman C.A., Wohlberg B. Plug-and-play priors for model based reconstruction. Proceedings of the 2013 IEEE Global Conference on Signal and Information Processing.

[32] Pendu M.L., Guillemot C. (2023). Preconditioned Plug-and-Play ADMM with Locally Adjustable Denoiser for Image Restoration. SIAM J. Imaging Sci..

[33] Cascarano P., Piccolomini E.L., Morotti E., Sebastiani A. (2022). Plug-and-Play gradient-based denoisers applied to CT image enhancement. Appl. Math. Comput..

[34] Kamilov U.S., Mansour H., Wohlberg B. (2017). A plug-and-play priors approach for solving nonlinear imaging inverse problems. IEEE Signal Process. Lett..

[35] Hurault S., Kamilov U., Leclaire A., Papadakis N. (2023). Convergent Bregman Plug-and-Play Image Restoration for Poisson Inverse Problems. arXiv.

[36] Romano Y., Elad M., Milanfar P. (2017). The little engine that could: Regularization by denoising (RED). SIAM J. Imaging Sci..

[37] Combettes P.L., Pesquet J.C. (2011). Proximal splitting methods in signal processing. Fixed-Point Algorithms for Inverse Problems in Science and Engineering.

[38] Chierchia G., Chouzenoux E., Combettes P.L., Pesquet J.C. The Proximity Operator Repository. http://proximity-operator.net/index.html.

[39] Chan S.H., Wang X., Elgendy O.A. (2017). Plug-and-Play ADMM for Image Restoration: Fixed-Point Convergence and Applications. IEEE Trans. Comput. Imaging.

[40] Sun Y., Wu Z., Xu X., Wohlberg B., Kamilov U.S. (2021). Scalable plug-and-play ADMM with convergence guarantees. IEEE Trans. Comput. Imaging.

[41] Nair P., Gavaskar R.G., Chaudhury K.N. (2021). Fixed-Point and Objective Convergence of Plug-and-Play Algorithms. IEEE Trans. Comput. Imaging.

[42] Cascarano P., Benfenati A., Kamilov U.S., Xu X. (2024). Constrained Regularization by Denoising with Automatic Parameter Selection. IEEE Signal Process. Lett..

[43] Immerkaer J. (1996). Fast noise variance estimation. Comput. Vis. Image Underst..

[44] Lin T., Ma S., Zhang S. (2015). On the Global Linear Convergence of the ADMM with MultiBlock Variables. SIAM J. Optim..

[45] Bevilacqua M., Roumy A., Guillemot C., Alberi-Morel M.L. Low-complexity single-image super-resolution based on nonnegative neighbor embedding. Proceedings of the 23rd British Machine Vision Conference (BMVC).

[46] Kodak Lossless True Color Image Suite. https://r0k.us/graphics/kodak/.

[47] Zhang K., Zuo W., Gu S., Zhang L. Learning Deep CNN Denoiser Prior for Image Restoration. Proceedings of the IEEE Conference on Computer Vision and Pattern Recognition (CVPR).

[48] Dabov K., Foi A., Katkovnik V., Egiazarian K. (2007). Image denoising by sparse 3-D transform-domain collaborative filtering. IEEE Trans. Image Process..

[49] Buades A., Coll B., Morel J.M. (2011). Non-local means denoising. Image Process. Line.

